# Fe_3_O_4_-TiO_2_ Thin Films in Solar Photocatalytic Processes

**DOI:** 10.3390/ma15196718

**Published:** 2022-09-27

**Authors:** Almudena Aguinaco, José M. Mánuel, Eduardo Blanco, Manuel Domínguez, Rocío Litrán, Juan J. Delgado, Milagrosa Ramírez-del-Solar

**Affiliations:** 1 Departamento Física de la Materia Condensada, Instituto de Microscopía Electrónica y Materiales (IMEYMAT), Universidad de Cádiz, 11510 Puerto Real, Spain; 2 Departamento Ciencias de los Materiales e Ingeniería Metalúrgica y Química Inorgánica, Instituto de Microscopía Electrónica y Materiales (IMEYMAT), Universidad de Cádiz, 11510 Puerto Real, Spain

**Keywords:** titania, magnetite, thin film, photocatalysis

## Abstract

The optical properties of 5wt% Fe_3_O_4_-TiO_2_ thin films were evaluated in detail with the aim of proposing a mechanism for solar photocatalytic processes and highlighting the advantages over the use of bare TiO_2_. The results showed that the incorporation of 5wt% Fe_3_O_4_ enhanced the optical properties by a redshift to a wavelength in the visible range, reducing the anatase/rutile band gap energy from 3.2 eV to 2.8 eV. Photoluminescence studies reveal a superior separation efficiency of photoexcited electron-hole pairs when Fe_3_O_4_ nanoparticles (NPs) are present in the photocatalyst. X-ray photoelectron spectroscopy spectra confirm the presence of Fe_3_O_4_ and existence of a chemical bonding between TiO_2_ and Fe_3_O_4_ NPs. Moreover, in this study, a mechanism of solar photocatalytic processes involving Fe_3_O_4_-TiO_2_ thin films is proposed and it is supported by experimental results. Finally, solar photocatalytic experiments were carried out, indicating that the effectiveness for the removal of the selected pharmaceutical is considerably improved when the composite material is used as catalyst. Furthermore, it was demonstrated that the photocatalytic activity of the prepared Fe_3_O_4_-TiO_2_ thin films depends on their thickness, achieving the highest pharmaceutical removal yields using the 2 µm thick sample. The stability and reusability of the catalyst was confirmed studying the photocatalytic activity over three cycles.

## 1. Introduction

In recent years, semiconductor photocatalytic processes have found wide applications in water treatment [1]. Titanium dioxide (TiO_2_) has been considered as the most widely used semiconductor oxide photocatalyst due to its attractive attributes, such as low cost, corrosion resistance, excellent chemical stability, non-toxicity and high photocatalytic activity [2]. However, the use of TiO_2_ nanoparticles (NPs) in solar photocatalysis for environmental remediation has some technical limitations, such as the high band gap of TiO_2_ (~3.2 eV) [3,4], close to UV spectral range. For this reason, this photocatalyst is triggered by near UV radiation. Thus, it takes advantage of only a small range of the solar spectrum (about 3–4%), which limits the potential of solar light as an energy source. Additionally, the photogenerated electron-hole pairs are liable to recombination, resulting in low quantum efficiency of TiO_2_ [5].

Moreover, the use of TiO_2_ as a photocatalytic material would increase the overall cost if it is utilized in powder form, which constitutes a drawback. This is so due to the necessity of performing an additional filtration stage in order to separate this powder from the water in solutions [6]. Thus, the separation of the semiconductor TiO_2_ catalyst after water treatment remains as the major obstacle towards the practicality of this technology [7]. In addition, nanoparticles, dispersed in the medium, can be dangerous due to their possible cytotoxic and inflammatory effects [8,9].

In order to overcome these issues, there are several studies in the literature in which Fe_3_O_4_-TiO_2_ nanoparticles systems have already been investigated. These studies revealed that the presence of Fe_3_O_4_ enhance the photocatalytic activity of TiO_2_ and that modified TiO_2_ NPs can be separated from water by means of an external magnetic field [10,11,12,13,14]. It is important to note that composites/heterojunction catalysts enhance the electron-hole separation and prevent charge back transfer [15,16].

However, we consider that modifying TiO_2_ with Fe_3_O_4_ would not lead to a feasible solution, since: (1) the suspended NPs tend to aggregate, especially when high concentrations are used [6] and furthermore, (2) the use of magnetic separation is complicated in real water treatment applications.

Therefore, a more realistic proposal would consist of depositing thin film coatings onto a substrate material [17]. In this sense, it is important to note that there are only few studies about the immobilization of Fe_3_O_4_-TiO_2_ NPs on substrates as thin films, being very few of them those related to its use in photocatalytic processes. For instance, Wei et al. [18] prepared Fe_3_O_4_-TiO_2_ composite films to enhanced the ability to store energy under low operating electric field. Zhang et al. [19] used Fe_3_O_4_ nanoparticles and molecularly imprinted TiO_2_ in the fabrication of a novel photoelectrochemical biosensor. Yeh et al. [20] evaluated the antimicrobial efficiency of immobilized Fe_3_O_4_/TiO_2_ powder on glass substrate. Nevertheless, to the best of our knowledge, no studies have been reported on the use of Fe_3_O_4_-TiO_2_ thin film for solar photocatalytic processes. Therefore, the current work represents a highly innovative study, which could open a new field of application for this particular material.

Thus, the purpose of this paper is to develop a Fe_3_O_4_-TiO_2_ composite material with high photocatalytic activity and good stability and their immobilization on substrates. In this study, thin films are prepared using a “sandwich” type structure [21], which prevents the release of NPs to the medium. Furthermore, the photocatalytic efficiency is evaluated by studying the degradation of an antibiotic, sulfamethoxazole (SFX), in the presence of these films. SFX is a sulphonamide antibiotic, which is one of the common pollutants in surface water [4,22,23,24,25].

The main motivation for using an antibiotic as a contaminant model in solar photocatalytic experiments is that conventional methods implemented in wastewater treatment plants are not efficient enough to remove pharmaceutical contaminants [25,26,27]. Therefore, these pollutants can arrive in rivers, lakes, seas, aquifers and occasionally, they can be found in tap water, although in very small quantities [28]. The presence of pharmaceutical compounds in surface and wastewater, all over the world, involves a significant risk for aquatic ecosystems and for all living beings and the environment [29,30,31]. Considering the effects and consequences to the environment, animals and plants, and regarding the frequency with which pharmaceuticals have been detected in water, it is expected that these types of pollutants will be candidates for future regulations.

Therefore, the research on alternative, resultative and cost-efficient technologies is a field of great interest for all the technological and scientific community.

## 2. Materials and Methods

### 2.1. Chemicals

Iron (II) chloride (FeCl_2_), iron (III) chloride (FeCl_3_) and ammonium hydroxide (NH_4_OH) were used to prepare magnetite (Fe_3_O_4_) NPs. Tetrabutil-ortotitatanate (TBOT) C_16_H_36_O_4_Ti (98%), acetylacetone C_5_H_8_O_2_ (99.5%), TiO_2_ nanoparticles (P25, 99.5%) and ethanol C_2_H_6_O (99.5%) were used to fabricate sandwich-type structure thin films. SFX and T-Butanol were used in the photocatalytic experiments. These experiments were carried out using MilliQ water.

Acetylacetona and ethanol were received from Merck and Panreac (ITW companies), respectively. The rest of reagents were purchased from Sigma-Aldrich.

### 2.2. Preparation of Fe_3_O_4_-TiO_2_ Thin Film Photocatalyst

#### 2.2.1. Synthesis of Magnetite Nanoparticles

Magnetite (Fe_3_O_4_) nanoparticles have been prepared following a classical bottom-up chemical route based on a co-precipitation reaction. With the aim to obtain the mixed (II and III) iron oxide, we have started from Fe^2+^ and Fe^3+^ precursor salts, FeCl_2_ and FeCl_3_, respectively, and we have promoted the controlled co-precipitation of both salts. Briefly, 0.99 g of FeCl_2_ and 2.76 g of FeCl_3_ are dissolved in 100 mL of MilliQ water. The solution is placed in a three-necked flask and heated at 80 °C under reflux and with magnetic stirring, in a nitrogen atmosphere, in order to avoid the formation of non-magnetic iron oxide phases. After heating the solution for 10 min, 10 mL of 25% ammonium hydroxide solution is swiftly added to the flask, promoting the fast and homogeneous nucleation step to form the magnetite cores. The solution is still heated, keeping the temperature at 80 °C, for 30 additional minutes, in which the previously formed nuclei continue growing. Once the solution was cooled down to room temperature, it was filtered three times (using a 0.1 μm Millipore membrane), adding ethanol, in order to eliminate the excess of precursor species and/or residues from the reaction. As result, we obtained a magnetic black powder composed by magnetite NPs.

#### 2.2.2. Preparation of Fe_3_O_4_-TiO_2_ Nanoparticles

For preparation, 0.15 g of synthetized Fe_3_O_4_ NPs were initially dispersed in 10 mL deionized water and kept under ultrasonic irradiation for 30 min. Throughout this procedure, a stable ferrofluid was obtained. Next, the dispersion was added, drop by drop, to 3 g of TiO_2_ NPs, forming a slurry that was finally dried at 110 °C for 24 h. Finally, samples were annealed at 400 °C for 30 min. This processing would lead to a 5wt% load of Fe_3_O_4_ NPs in TiO_2_. This concentration value (around 5wt%) has shown to give the best optical and structural properties as well as superior photocatalytic activity in FeTiO_2_ samples [32,33,34].

#### 2.2.3. Preparation of Photocatalyst Films

Photocatalyst coatings on flat glass supports were prepared according to a multilayer approach proposed in previous works by scientists of our research group [4,21]. According to the methodology, the structure of the photocatalyzer responds to a “sandwich” approach consisting of two very thin anatase films (~50 nm), obtained by sol gel [35], delimiting a thicker layer obtained from a suspension of TiO_2_/Fe_3_O_4_ nanoparticles.

Anatase films were deposited from a precursor sol prepared by hydrolysis of titanium n-butoxide (TBOT) in a solution containing acetylacetone, ethanol and acidified water (HNO_3_, pH = 1) in a molar ratio TBOT:acetylacetone:H_2_O:EtOH of 1:0.5:2:35 as described in Blanco et al. (2015) [35]. Dip coating was performed from aged sol at precisely controlled withdrawal speed (100 mm/min) in a homemade apparatus on a microscope slide and the resulting film was subsequently dried at 150 °C for 30 min and annealed at 1°/min until 500 °C, in order to ensure that TiO_2_ films is formed by small anatase crystallites while preserving a certain porosity level in the films which provides high surface areas [36].

For the central part of the sandwich coating, a suspension of the Fe_3_O_4_-TiO_2_ nanoparticles in an acetylacetone-ethanol solution (4:1 vol ratio, 40 g/L) was prepared by using high power ultrasounds (20 KHz). Films were dip coated on the top of the previous anatase film at 100 mm/min, layer by layer. After every five depositions, the layers are dried at 150 °C for 30 min and the process is repeated until reaching the total number of nanoparticulated layers are deposited.

Finally, the coating was sealed with a new single anatase thin film prepared as described before. The final photocatalyst is obtained after annealing in air for 1 h at 400 °C. For comparison, a similar non-doped “sandwich” coating was also prepared using a suspension of TiO_2_ NPs a precursor of the central layer. The resulting film was used as a reference.

### 2.3. Photocatalyst Characterization

Magnetite NPs were studied by Transmission Electron Microscopy (TEM) and Scanning-Transmission Electron Microscopy (STEM), using a JEOL JEM-2100 EX TEM and a FEI TALOS F200X STEM microscope, with thermionic effect and field effect electron emission guns, respectively, both working at 200 kV. Both microscopes are located at the facilities of Servicios Centrales de Investigación, Ciencia y Tecnología (SC-ICYT) of the University of Cádiz (UCA). NPs were deposited in TEM holey carbon grids according to a previously stablished method [37]. Dynamic Light Scattering (DLS) was utilized for measuring the hydrodynamic NP size. Measurements were carried out at 25 °C, using a Malvern Zetasizer Nano-ZS (“Malvern Instruments”, Worcestershire, U.K.), with a 1 cm path cell. This equipment also allows for the Z-potential to be measured which evaluate colloidal solutions stability, as well as NP surface charge.

In order to study the effect of incorporating Fe_3_O_4_ NPs on the optical properties of TiO_2_ NPs and estimate their energy band gap, UV-Vis absorbance and diffuse reflectance measurements were carried out using an Agilent Cary 5000 UV-Vis-NIR double-beam spectrophotometer. The spectra in the 200 to 800 nm range were registered in an integrating sphere. The resulting diffuse reflectance spectra were transformed into apparent absorption spectra using the Kubelka-Munk function (*F*(*R*)). The direct and indirect optical band gap of the materials was determined through the construction of Tauc plots by plotting (*F*(*R*)*hν*)*^n^* against (*hν*), with *n* = 2 or *n* = ½, for direct and indirect transitions, respectively. The optical band gap was obtained by extrapolating the linear part of this plot to the energy axis. Furthermore, the photoluminescence spectra were obtained using a Fluorolog-QM spectrometer (Horiba) equipped with a continuous 75 W Xenon arc lamp, double monochromators at the excitation and the emission and a photomultiplier tube detector (PMT-920IS). Temperature controlled experiments were performed with a cryostat (CS204-FMX-1SS, ARSCRYO) that can be operated between 4 and 350 K.

X-ray photoelectron spectroscopy (XPS) was recorded using a Kratos Axis Ultra DLD spectrometer. XPS measurements were performed using a monochromatic Al Kα radiation at 1486.6 eV and X-ray power of 150 W. The analyser was operated in constant analyser energy transmission (CAE) mode, with pass energy of 80 eV and 20 eV for the survey scan spectrum and for high-resolution core levels, respectively. Surface charging effects were compensated by making use of the Kratos coaxial neutralization system. The binding energy (BE) scale was calibrated with respect to the C 1 s signal at 284.8 eV. XPS data analysis was performed by using CasaXPS Software, version 2.3.19rev1.1 m (Neal Fairley, Casa Software Ltd., Teignmouth, Devon, UK).

Morphology and thickness of prepared thin films was followed by Scanning Electron Microscopy (SEM), recording cross-section images obtained using the secondary electron detector of a FEI Nova NanoSEM 450 scanning microscope (also located at the UCA-SC-ICYT facilities), with field effect electron emission gun, working at a voltage and a distance of 20 kV 4.6 mm, respectively.

Atomic Force Microscopy (AFM) images were obtained with a DI Veeco Nanoscope Multimode IIIa equipment using Bruker TESP-V2 probes and working in tapping mode. AFM images were processed with the open-source software Gwyddion [38].

### 2.4. Photocatalytic Experiments

Experiments were carried out using a custom-made photocatalytic system, consisting of a magnetic-stirred reactor exposed to sunlight, equipped with a recirculating peristaltic pump (Selecta Percom N-M) and coupled to an Avantes fiber optic UV-Vis-NIR spectrometer (Ocean Optics DT-Mini-2GS light source) that allows continuous monitoring [4].

For each experiment, 20 mL of an aqueous solution of SFX was charged into the photoreactor (5.6 mm diameter). SFX initial concentration was 5 ppm. A Fe_3_O_4_-TiO_2_ thin film (2.5 × 2.8 cm^2^) was also place into the reactor. Experiments were conducted at Campus de Puerto Real, University of Cadiz (Spain, local latitude 36°31′ N) on sunny days. A radiometer UV34 (PCE Ibérica, Spain) was used to measure the intensity of solar radiation.

Absorbance measurements were determined using a UV-Vis-NIR fibre optic spectrometer and its evolution over radiation dose was monitored due to a peristaltic pump that recirculated (6 mL/min) the solution treated by solar photocatalysis.

Cumulative UV dose was calculated as shown in Equation (1).

(1) $$Q_{\mathrm{UVn}}=Q_{\mathrm{UVn}-1}+\sum \left({\mathrm{UV}}_{n}\cdot \Delta t\right)\frac{V_{i}}{V_{T}}$$

where Q_UVn_, Q_UVn−1_ is the cumulative irradiated UV energy received at times n and n − 1, respectively; Δt_n_ the time interval between two sampling times; UV_n_ the average incident radiation for each time interval (W/m^2^); Vi the illuminated volume of the reactor and V_T_ the total volume of the reactor.

Experimental results can also be expressed using *t*_30W_ (see Equation (2)). It is a normalized value that indicates the time spent on the experiment in the hypothetically situation of a constant UV radiation intensity of 30 W/m^2^, during its development [39]. A UV intensity of 30 W/m^2^ resembles conditions around a sunny noon in the vicinity of Cádiz.

(2) $$t_{30Wn}=t_{30Wn-1}+\sum \left(\frac{UV_{n}}{30}\cdot \Delta t\right)\frac{V_{i}}{V_{T}}$$

where *t*_30W*n*_ *t*_30W*n*−1_ are the normalised illumination time of samples at times *n* and *n* − 1, respectively.

## 3. Results and Discussion

### 3.1. Fe_3_O_4_ and Fe_3_O_4_-TiO_2_ NPs Morphology, Size, Crystallinity and Composition

**Figure 1:**
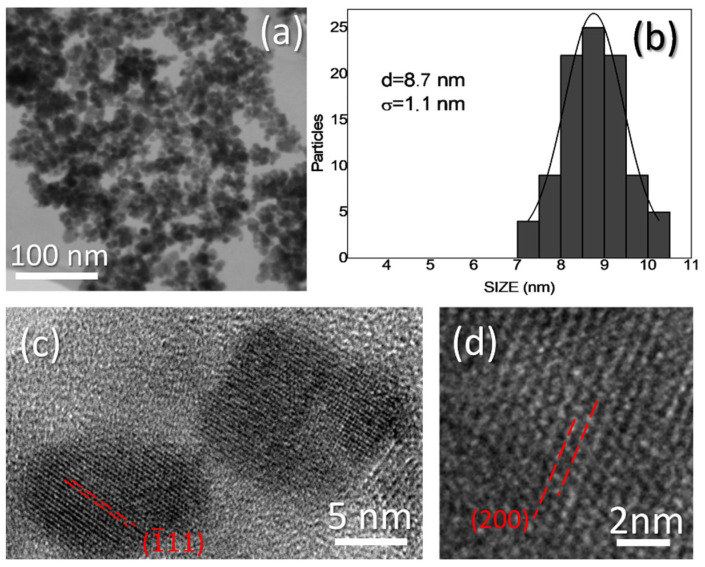
(**a**) SEM micrograph, (**b**) particle size distributions and (**c**,**d**) HRTEM micrographs of magnetite NPs.

Figure 1a corresponds to a SEM micrograph, showing a general view of the synthetized magnetite NPs. This image and others alike were utilized as a first indication of the quality of the synthetized NPs and to calculate the average particle size. Micrograph reveals the formation of relatively small nanoparticles with homogeneous size distribution. Although the strong magnetic character of the material leads to the NP aggregation, the higher atomic number of iron gives a good contrast that allows distinguishing the formation of approximately spherical particles whose concentration is high enough for performing a statistical study on the size distribution. In this sense, the histogram of this magnitude is presented in Figure 1b. As it can be observed, the experimental particle size distribution fits well with a Gaussian distribution. This fit indicates a NP average size of 8.7 nm with a small standard deviation (1.1 nm). NP crystalline structures can be observed in High Resolution TEM (HRTEM) images, as the ones in Figure 1c and, at higher magnification, Figure 1d. The averaged experimental distances between crystalline planes are 3.00 and 3.43 Å, respectively. These distances best agree with those of the theoretical ones for ($\bar{1}$11) and (200) planes of magnetite [40]. These measured planes have been labelled and marked in the figure.

On the other hand, Table 1 shows the average hydrodynamic size, PDI (polydispersity index) and Z-Potential values for magnetite NPs. As expected, the hydrodynamic size determined by the DLS technique is significantly higher in comparison with the one obtained by TEM. This effect is assigned to the presence of extra hydrate layers in aqueous medium. Moreover, some agglomeration of the nanoparticles in aqueous solutions is expected due to the magnetism of magnetite NPs. However, this average hydrodynamic size, together with the relatively high value of the Z-potential, indicates the NPs colloidal stability. In addition, the positive value of the Z-potential suggests the presence of positive charge at the NP surface, which could promote subsequent crosslinking with the matrix.

**Figure 2:**
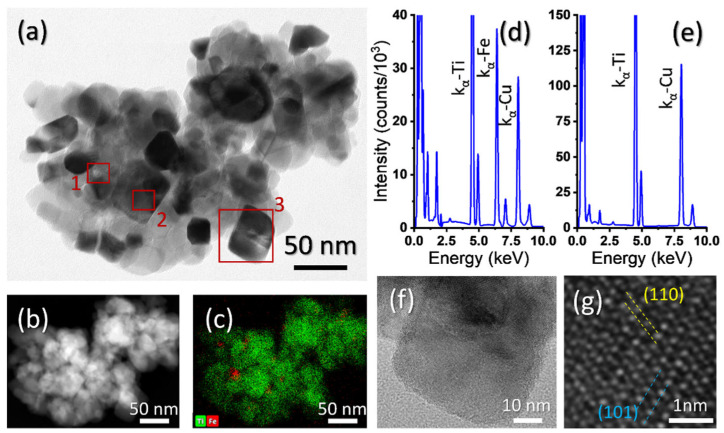
(**a**) TEM-BF micrograph of magnetite NPs-doped TiO_2_. (**b**) HAADF image and (**c**) EDX map of the same material. EDX point spectrum of region of titanium oxide (**d**) containing a magnetite NP and (**e**) without NP. (**f**,**g**) HRTEM micrographs of anatase TiO_2_.

In order to find out whether or not the utilized TiO^2^ doping process produce a material in which the magnetite NPs are actually imbibed in the titanium oxide matrix, small volumes of Fe_3_O_4_-TiO_2_ were deposited on holey-carbon TEM grids, for their compositional study. One of these volumes is presented in Figure 2a, which corresponds to a TEM micrograph obtained in Diffraction Contrast-Bright Field (BF) mode. The darker areas correspond to orientations of the material (TiO_2_) along which the electron beam is parallel to high atomic density crystallographic planes. This doped TiO_2_ volume was later observed using STEM techniques, such as High Angle Annular Dark Field (HAADF) and Energy Dispersive X-ray spectroscopy (EDX), as presented in Figure 2b,c. HAADF images intensity highly depends on the atomic mass and thickness of the material. Therefore, and taking into consideration the small size of the NPs and roughness and irregularity of the TiO_2_ volume under study, it is difficult to distinguish these NPs in HAADF images. EDX spectra maps, on the other hand, easily revealed the positions of such particles in the titanium oxide matrix, as it can be observed in Figure 2c. In this map, red spots correspond to Fe characteristic X-rays emitted from the material, and green dots correspond to Ti-signal. Weaker Fe signals from the volume could be explained by different particle sizes or, more probably, because of the high possibility of the NP being totally contained inside the TiO_2_ volume. This was corroborated by EDX point spectra taken from the NPs. Figure 2d shows one of such spectra, obtained from the region “1” indicated in Figure 2a with a red square. This spectrum presents a Fe-peak, corresponding to the NP, and a Ti-peak (since the STEM probe interacts with a certain volume surrounding the NP). EDX point spectrum in Figure 2e, taken from the region labelled as “2” in Figure 2a, proves that the Fe-peak in the previous spectrum is not due to sample contamination. Cu-peak in both spectra comes from the Cu mesh of the utilized TEM grid. It can be concluded, thus, from this EDX map, that the utilized process indeed produces a Fe_3_O_4_-TiO_2_ slurry, with NPs randomly distributed in the TiO_2_. HRTEM images were obtained in order to investigate possible changes in the TiO_2_ structure. In this sense, images such as the one in Figure 2f (obtained from the region labelled as “3” in Figure 2a) and, at higher magnification, 2g allow measuring averaged lattice distances of 2.36 and 3.52 Å, which are in good agreement well with the theoretical distances for (110) and (101) for the atomic planes of the anatase phase of TiO_2_ [41]. The dashed lines in the figure are representative of the indicated lattice distances. Neither of the HRTEM images recorded shows, in any case, a change in the TiO_2_ crystalline structure, indicating the absence of a possible phase change or measurable stress due to the introduction of NPs.

### 3.2. Thin Film Characterization

#### 3.2.1. Optical Properties: UV-Vis Spectra, Bandgap Energy and Photoluminescence Studies

**Figure 3:**
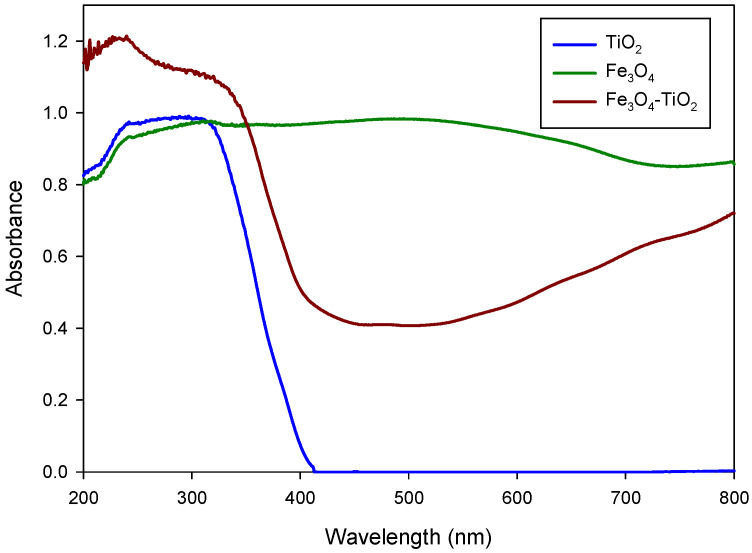
UV-vis-NIR absorption spectra of TiO_2_,Fe_3_O_4_-TiO_2_ and Fe_3_O_4._

The catalysts were deposited in the form of thin films. Figure 3 shows the UV-Vis absorption spectra of Fe_3_O_4_-TiO_2_ and TiO_2_ photocatalysts, where it is possible to assign some common features in the spectral range beyond 300 nm, despite the strong absorption of the substrate at this wavelength. TiO_2_ spectrum shows a strong absorption corresponding to the absorption edge of semiconductor TiO_2_ below 400 nm but, in the spectral range above 420 nm, shows very little absorption. Conversely, spectrum corresponding to Fe_3_O_4_-TiO_2_ sample exhibits a broad absorption across the whole visible spectrum, as was previously described in the literature [42].

Optical band gap energy determination of the doped catalyst and its components were evaluated from UV-Vis diffuse reflectance spectra by applying the Kubelka-Munk (K-M) function [21,43]. The K-M function, *F*(*R*), establishes a relationship between absorbed, scattered and diffused reflected light by the sample, and is defined as:

(3) $$F\left(R\right)=\frac{{\left(1-R\right)}^{2}}{2R}$$

where *R* is the wavelength dependent sample diffuse reflectance, measured with the spectrophotometer integrating sphere. The use of this model for this kind of coating has been justified by considering the granular structure for these optically rough films [36].

Thus, the Tauc method can be applied to the function (F(R)*E)^m^ versus the photon energy, E, with m = 1/2 or m = 2 for indirect or direct interband transitions, respectively. The (F(R)*E)^m^ function will present a linear region for E values close to E_g_ where the relationship (F(R)*E)^m^ = k(E − E_g_) applies. In this way, the gap energy of Fe_3_O_4_-TiO_2_ thin film, E_g_, can be obtained from the extrapolation to zero of the linear regression in this region of the plot. Both direct and indirect bandgaps (Table 2) calculated in this way from plots using m = 1/2 and m = 2, respectively, are collected in Table 2.

It can be noticed that materials shown in Table 2 exhibit an indirect bandgap smaller than the direct band gap, suggesting that the fundamental bandgap for these materials could be considered as an indirect bandgap [44,45]. The direct or indirect nature of the bandgap transition is an essential parameter of semiconductors for photocatalytic applications. Thus, when a semiconductor has an indirect bandgap and the electric dipole transition from valence band maximum to conduction band minimum is allowed, then the electron-hole pairs recombine with a lower probability [46].

As it can be seen in Table 2, the calculated indirect bandgap of TiO_2_ is nearly 3.2 eV but it is reduced when Fe_3_O_4_ NPs are included in the material. The small indirect energy gap obtained for magnetite NPs is consistent with the fact that Fe_3_O_4_-TiO_2_ materials present absorption in the visible spectrum region.

This lower bandgap energy of the doped material is due to the fact that the presence of Fe_3_O_4_ NPs introduce a series of localized occupied states into the bandgap of TiO_2_ lattice [47].

Narrowing the bandgap of TiO_2_ is an important strategy to enhance its photocatalytic activity. It has been shown that TiO_2_ surface loading with Fe_3_O_4_ nanoparticles can be used to enhance the electron-hole pairs generation efficiency to stimulate photocatalytic reaction [48].

**Figure 4:**
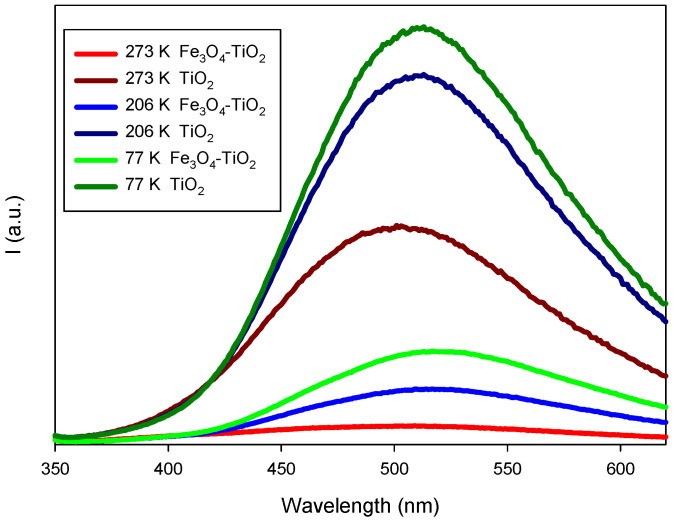
Photoluminescence spectra of TiO_2_ and Fe_3_O_4_-TiO_2_ thin films, obtained at excitation wavelengths of 325 nm.

PL studies were conducted to elucidate the effect of the electron-hole annihilation on the photocatalytic properties. The steady state photoluminescence spectra of synthesized photocatalysts were registered using an excitation wavelength of 325 nm and different temperatures (77,206 and 273 K). Our results showed (Figure 4) that pure TiO_2_ exhibits the highest PL emission intensity, what can be directly related to a high electron-hole recombination rate [49]. PL intensity of Fe_3_O_4_-TiO_2_ was significantly smaller, indicating a noteworthy lower recombination rate, i.e., a superior separation efficiency of the photoexcited electron-hole pairs [49]. Thus, the addition of Fe_3_O_4_ nanoparticles on the surface of TiO_2_ can significantly reduce the electron-hole pairs recombination by accepting the electrons from TiO_2_ [48].

#### 3.2.2. Composition, Structure and Morphology

**Figure 5:**
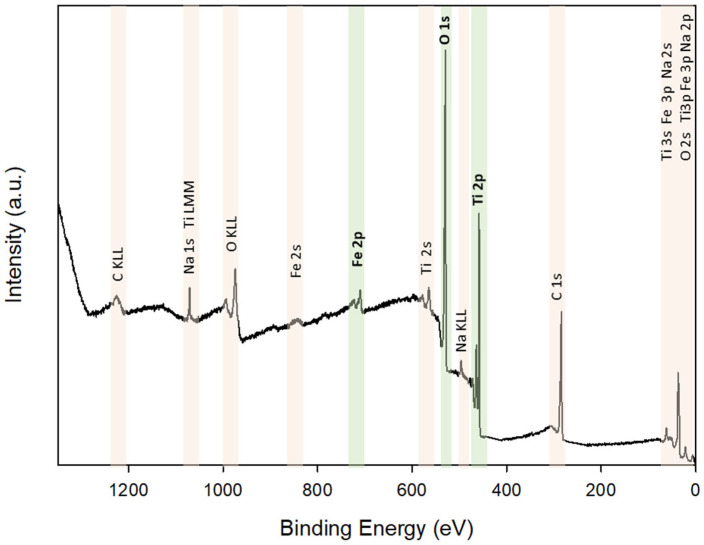
XPS survey scan spectrum of Fe_3_O_4_-TiO_2_ thin film.

X-ray photoelectron spectroscopy was carried out in order to investigate the electronic features and elemental composition of the prepared materials. Figure 5 shows the survey scan XPS spectrum of Fe_3_O_4_-TiO_2_ thin films, which contains O 1 *s*, Ti 2*p* and Fe 2*p* peaks.

**Figure 6:**
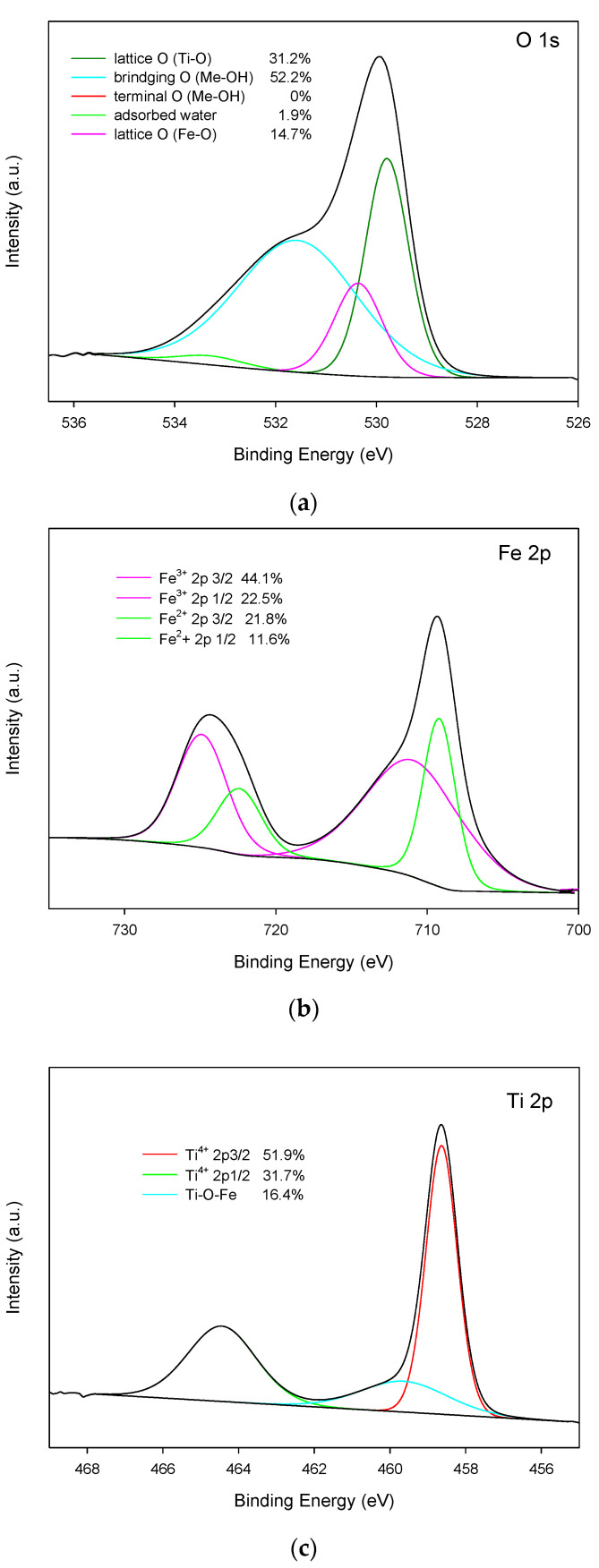
XPS spectra of curve fitting for: (**a**) O 1*s*; (**b**) Fe 2*p*; (**c**) Ti 2*p*.

High Resolution XPS signal and their deconvolutions are presented in Figure 6a–c:

O 1*s*: a high-resolution O 1*s* XPS spectrum is deconvoluted into four peaks.

From those computationally obtained peaks, the ones centred at energies of 529.8 and 530.4 eV can be assigned to the oxygen in the lattice Ti-O and to the oxygen in Fe-O, respectively. The peak located at 531.6 eV is related to the oxygen atoms in ‘bridging’ hydroxyl groups that are bound to titanium or iron atoms (Me-OH) [50]. Two types of hydroxyl groups are defined in the literature [51,52]: one of them, referred to as a ‘bridging’ hydroxyl, is attributed to O_2_^−^ anions in the bulk structure of the material. The other one, which called ‘terminal’ and resides at an acidic site, is attributed to chemisorbed OH groups on the sample surface. As can be deduced from spectrum shown in Figure 6a, no terminal hydroxyl groups were detected in the fabricated material.

The O atom in the bridge OH should be energetically favourable to attract positive holes, because it has negative charge. Consequently, a certain fraction of trapped holes probably release ^•^OH into bulk solution [53].

Fe 2*p*: in order to confirm the presence of Fe_3_O_4_, the elemental and valence state analysis were performed using XPS. Figure 6b shows the core-level XPS spectra of Fe 2*p* corresponding to Fe_3_O_4_. The peaks located at 711 and 724.6 eV are associated to Fe(III) 2*p*_3/2_ and Fe(III) 2*p*_1/2_, respectively. Peaks at 709.3 eV and 722.5 eV can be assigned to Fe (II) 2*p*_3/2_ and Fe(II) 2*p*_1/2_, respectively. These results are in agreement with Fe 2p XPS spectrum of Fe_3_O_4_ reported in the literature [54]. Moreover, the area ratio of Fe^3+^/Fe^2+^ is about 0.50, which is consistent with the stoichiometry of Fe_3_O_4_ [55].

Ti 2*p*: the core-level XPS spectra of Ti 2*p* is fitted with three peaks. Peaks located at 458.6 eV and 464.4 eV can be assigned to Ti^4+^, confirming the main valence state of + 4 for Ti. Moreover, the energy difference between Ti2*p*_1/2_ and Ti2*p*_3/2_ is 5.8 eV, which is associated to the TiO_2_ standard binding energy value [56]. Additionally, the calculated peak that corresponds to the Ti in the Ti-O-Fe bond is centred at an energy of 459.7 eV. It is worth noting that the existence of this peak, which is a necessary result of the deconvolution of the experimental XPS signal, is proof of the existence of a chemical bonding between TiO_2_ and Fe_3_O_4_ NPs [50,56].

**Figure 7:**
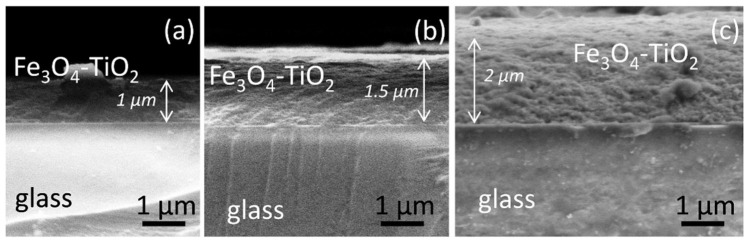
SEM micrographs of the prepared photocatalysts: (**a**) 1 µm:Fe_3_O_4_-TiO_2_; (**b**) 1.5 µm:Fe_3_O_4_-TiO_2_; (**c**) 2 µm:Fe_3_O_4_-TiO_2_.

Once the composition of the Fe_3_O_4_-TiO_2_ thin film was analysed by XPS, and taking into account the expected influence of the thickness in the photocatalysis experiments, the photocatalysts with 5, 10 and 15 layers deposited were prepared, and SEM in the cross section was performed to get thickness measurements. Samples were called “1 µm:Fe_3_O_4_-TiO_2_”, “1.5 µm:Fe_3_O_4_-TiO_2_” and “2 µm:Fe_3_O_4_-TiO_2_”, according to their nominal thicknesses. SEM micrographs of the prepared photocatalysts are shown in Figure 7. These images allowed to obtain the experimental thicknesses of the Fe_3_O_4_-TiO_2_ layer for each sample: (0.97 ± 0.03), (1.51 ± 0.04) and (1.99 ± 0.02) µm, respectively. These experimental values are in very good agreement with the nominal ones.

SEM images in Figure 7 indicates, beside the spatial scale, the expected 1, 1.5 and 2 μm thicknesses for each case. Note that, due to sample orientation and the clivationmethod followed in order to observe the materials in the SEM microscope, images in Figure 7 shows not only the layers profiles, but also, in the cases of Figure 7b and c, the surface of the preparations. Such surface can be distinguished from the profile by the intensity contrast in the image.

**Figure 8:**
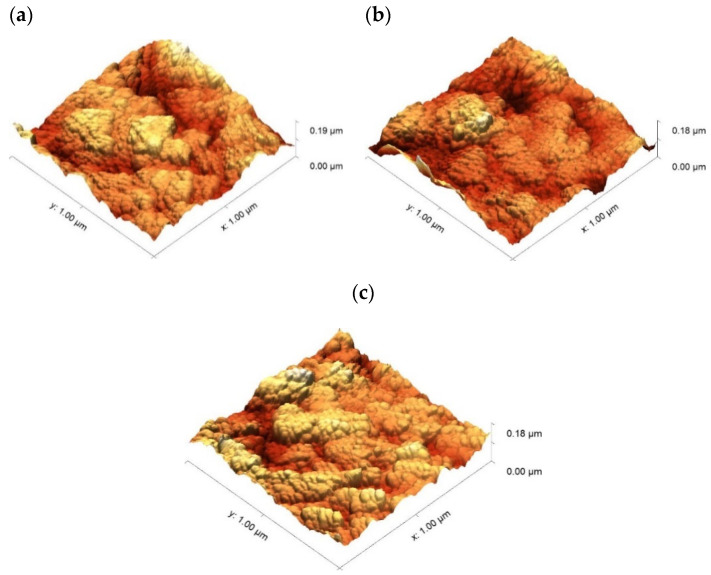
Three-dimensional AFM images of Fe_3_O_4_-TiO_2_ films with thicknesses of: (**a**) 1 µm:Fe_3_O_4_-TiO_2_; (**b**) 1.5 µm:Fe_3_O_4_-TiO_2_; (**c**) 2 µm:Fe_3_O_4_-TiO_2_.

Surface roughness of the titanium oxide layer for these three photocatalysts samples (1 µm:Fe_3_O_4_-TiO_2_, 1.5 µm:Fe_3_O_4_-TiO_2_ and 2 µm:Fe_3_O_4_-TiO_2_) was also determined, using atomic force microscopy (Figure 8). Measurements were repeated in three different locations on their surfaces. These measurements and their averaged values are collected in Table 3. The values in this table reveal that, the appearance of surface, in all cases, is similar and quite homogeneous, having a roughness which seems to be independent of the layer thickness. It means that this method of fabrication would allow using even thicker layers without a modification in the surface roughness, which is an important factor that influences the physical behaviour of TiO_2_ -based thin films [57].

### 3.3. Proposed Mechanism

Based on Equations (4) and (5) and using the experimental band gap energies (see Table 2), the valence band energy, *E_VB_*, of TiO_2_ and Fe_3_O_4_ can be calculated to be 2.91 eV and 1.74 eV, respectively.

(4) $$E_{CB}=\chi -E^{c}-0.5E_{g}$$

(5) $$E_{g}=E_{VB}-E_{CB}$$

being the absolute electronegativities (χ) of TiO_2_ and Fe_3_O_4_ 5.81 eV and 5.77 eV, respectively, and *E^C^* = 4.5 eV is the scaling factor relating the normal hydrogen electrode scale (NHE) to absolute vacuum scale [58].

**Figure 9:**
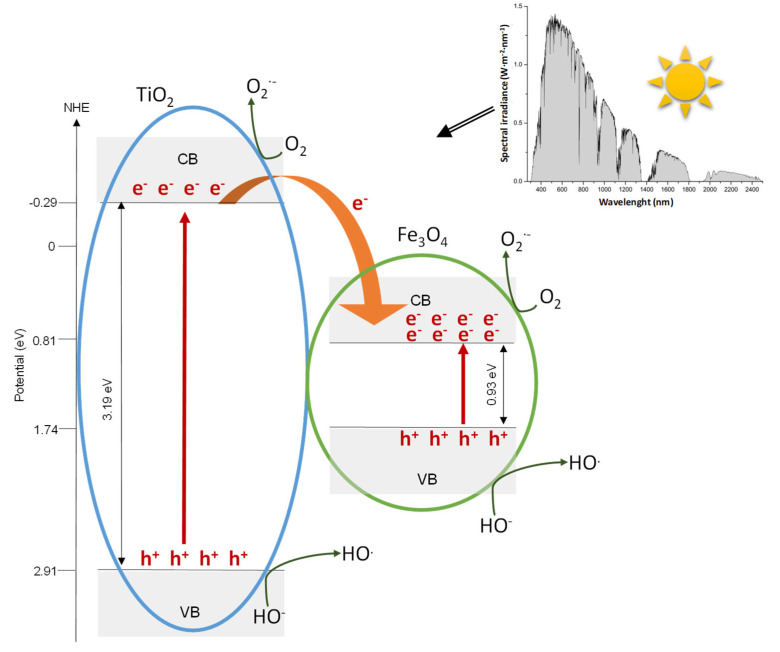
Proposed mechanism.

As can be seen in Figure 9, when Fe_3_O_4_-TiO_2_ is solar irradiated (see Solar spectrum shown in the embedded Figure [59]), the different steps that take place during the photocatalytic process are:

1. Electron-hole pairs are produced in TiO_2_ and Fe_3_O_4_ nanoparticles.
2. Electrons can be easily transferred from the conduction band (CB) of TiO_2_ to the CB of Fe_3_O_4_. It is a consequence of the positive energy difference between conduction bands of Fe_3_O_4_ and TiO_2_ (3.19 and 0.93 eV, respectively).
3. The transferred electrons would then migrate to the surface of Fe_3_O_4_ to further assist to oxygen reduction to form superoxide radical anion during photocatalysis [60].
4. Additionally, electrons in the conduction band of TiO_2_ will further react with molecular oxygen O_2_ dissolved to also form the superoxide radical anion O_2_^·−^ [61].
5. Superoxide radical anion could recombine to yield hydrogen peroxide that can react with Fe(II) generated by the electrons transferred from the TiO_2_ to Fe_3_O_4_, via a Fenton reaction, to generate ^·^OH and Fe(III) [62].
6. On the other hand, holes located in the valence band of TiO_2_ are transferred to the surface of TiO_2_ generating hydroxyl radicals because of the oxidation of water or surface hydroxyl species [60].
7. Radiation corresponding to wavelength range above 388 nm (E < 3.2 eV) is not energetic enough to excite TiO_2_ and generate electron-hole pairs, whereas Fe_3_O_4_ could be easily activated in this spectral range and produces charge carriers. Subsequently, the photogenerated electrons should be involved in the processes previously described in the step 3 of this proposed mechanism.
8. The holes that are accumulated in the valence band of Fe_3_O_4_ will react with OH^−^ species existing on the surface of the catalyst, producing reactive hydroxyl radicals (^·^OH).

**Figure 10:**
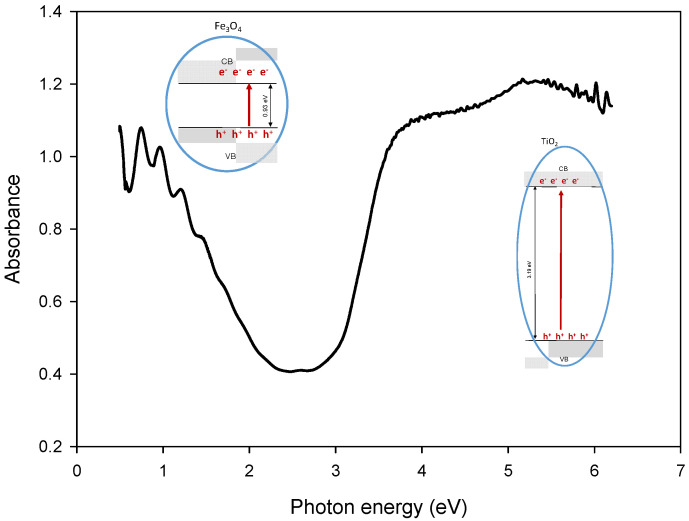
Absorbance spectra for Fe_3_O_4_-TiO_2_.

Most of the considerations made in the proposed mechanism could be supported by experimental results. Thus, PL studies revealed a superior separation efficiency of the photoexcited electron-hole pairs in Fe_3_O_4_-TiO_2_ NPs. Results obtained from the deconvolution of the high-resolution O 1*s* XPS show that positive holes generate hydroxyl radicals. In addition, as can be seen in Figure 10, absorption bands can be related to the energy levels proposed.

On the other side, the determined optical bandgap (Table 2) is originated by electronic coupling between the wide band gap of TiO_2_ and the narrow band gap of Fe_3_O_4_ [63].

Thus, via the proposed mechanism, it can be deduced that in Fe_3_O_4_-TiO_2_ material, not only the amount of photogenerated electron-hole pairs are increased, but also the separation efficiency of the photoexcited charges is enhanced. It is essential due to photocatalytic activity of photocatalysts which mainly depend on the extent of electron-hole pairs production and separation efficiency of these charge carriers [64].

### 3.4. Photocatalytic Experiments

Photocatalytic degradation of SFX is selected as a probe reaction to evaluate the photocatalytic activity of the so-prepared Fe_3_O_4_-TiO_2_ thin films. Solar photocatalysis experiments were performed treating 5 mg·L^−1^ aqueous solutions of SFX. The goal of photocatalytic experiments was not only to evaluate whether the incorporation of Fe_3_O_4_ NPs enhance the photocatalytic activity or not, but also to study the influence of thickness of the thin films on the SFX removal rates. Thus, in order to compare the effectiveness of Fe_3_O_4_-TiO_2_ films with that of pure TiO_2_, solar photocatalysis experiments were carried out using thin films with a similar thickness (~2 µm). Experimental results show that SFX follows first order kinetics according to Equation (6), in which t_30W_ can be calculated from experimental data by using Equation (2).

(6) $$ln\frac{C}{C_{0}}=-kt_{30W}$$

From this fitting process, pseudo-first order constants for TiO_2_ and Fe_3_O_4_-TiO_2_ thin film were calculated to be 6.95·10^−3^ min^−1^ and 1.35·10^−2^ min^−1^, respectively. As expected from the previously exposed optical characterization, when the composite material is used, the removal performance is considerably improved when solar photocatalytic processes are applied. Moreover, adsorption experiments indicated that SFX does not adsorb on the TiO_2_ and Fe_3_O_4_-TiO_2_ photocatalyst surface. These results are in agreement with the literature [22]. In addition, direct photolysis contribution is negligible. Thus, no elimination of SFX was observed when it was exposed to solar radiation, without the presence of the photocatalyst. In conclusion, it can be accepted that free radical oxidation is the main way of oxidation and, as was described in Section 3.3, hydroxyl radicals can be generated by different pathways.

In order to prove that the elimination is due to the presence of hydroxyl radicals, an experiment was carried out in the presence of T-butanol. T-Butanol is a very commonly used hydroxyl radical scavenger in aqueous matrices [24]. Results show that the presence of t-butanol completely inhibits the SFX removal.

**Figure 11:**
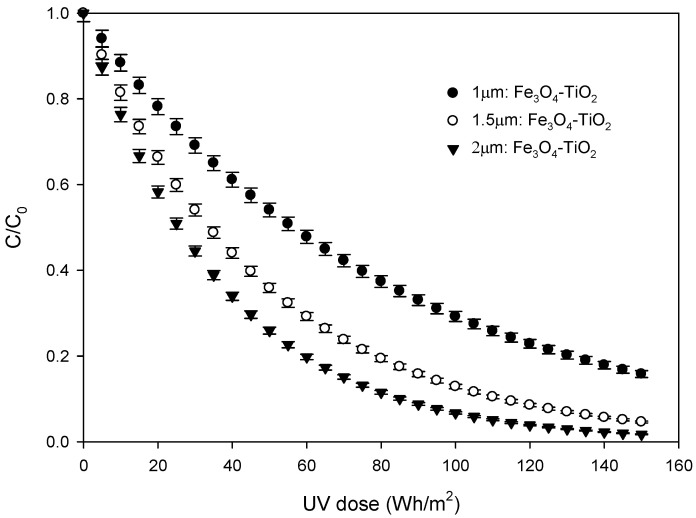
Degradation of SFX using thin films with different thickness.

On the order hand, in addition to the Fe_3_O_4_-TiO_2_ film with a thickness of 2 µm, thin Fe_3_O_4_-TiO_2_ films with thickness of around 1 and 1.5 µm were also prepared, as was mentioned above. Photocatalysis efficiency of these three samples is evaluated in Figure 11, in which the normalized concentration of SFX versus UV dose is represented. The presented results were calculated as an average value of the three repeated experiments per photocatalyst. Under solar irradiation, the concentration of the selected pharmaceutical evolves according to Equation (6) for all the cases and the calculated pseudo-first order constants are shown in Table 4. In view of the Figure 11 and Table 4, it is evident that a higher removal degree is achieved as the thickness of the Fe_3_O_4_-TiO_2_ film increases in the studied range.

As an example, for an accumulated radiation dose of 150 Wh/m^2^ (t_30w_ = 300 min), elimination yields of 84.2, 95.4 and 98.3% were reached, when 1 µm:Fe_3_O_4_-TiO_2_; 1.5 µm:Fe_3_O_4_-TiO_2_ and 2 µm:Fe_3_O_4_-TiO_2_ were used, respectively. Furthermore, differences observed when 1.5 µm:Fe_3_O_4_-TiO_2_ and 2 µm:Fe_3_O_4_-TiO_2_ samples were used are less significant than those observed when comparing the results obtained with the 1 and 1.5 µm thick samples, which may be due to the existence of a critical film thickness.

Taking into account that there is no elimination of SFX by adsorption or direct photolysis, the presence of light and photocatalyst are necessary.

Experimental results obtained (Figure 11) demonstrate that activity is governed by the catalyst film thickness. This result allows us to conclude that all the deposited material participates in the solar photocatalysis process.

Finally, in order to check the stability of the fabricated thin films, solar photocatalytic experiments were carried out reusing one of these films. Specifically, the 1.5 µm:Fe_3_O_4_-TiO_2_ photocatalyst was used in three consecutive reactions and no decrease in SFX elimination efficiency was observed.

## 4. Conclusions

The main conclusions of this work are:

Magnetite NPs and TiO_2_, as examined by electron microscopy, were of crystalline nature, and the utilized method to dope TiO_2_ resulted in imbibed magnetite NPs, randomly distributed in the titanium oxide. NPs size was (8.7 ± 1.1) nm. XPS confirmed the presence of Fe_3_O_4_ and the chemical bonding between TiO_2_ and Fe_3_O_4_ NPs.

The process to obtain the modified titanium oxide layer for the “sandwich” structure was well controlled, and tested for thicknesses between 1 and 2 µm.

The calculated indirect bandgap is reduced when Fe_3_O_4_ NPs are included in the material. The small indirect energy gap obtained for magnetite NPs is consistent with the fact that Fe_3_O_4_-TiO_2_ materials present absorption in the visible spectrum region.

The proposed mechanism of Fe_3_O_4_-TiO_2_ thin films in solar photocatalytic processes could be supported by experimental results. It was determined that, in Fe_3_O_4_-TiO_2_ material, not only are the number of photogenerated electron-hole pairs increased, but also the separation efficiency of the photoexcited charges is enhanced. Solar photocatalytic experiments evidenced the effectiveness improvement when modified thin films are used. Thus, pseudo-first order constants for TiO_2_ and Fe_3_O_4_-TiO_2_ 2 µm thin films were calculated to be 6.95·10^−3^ min^−1^ and 1.35·10^−2^ min^−1^, respectively.

Regarding the thickness of the fabricated thin films, AFM measurements revealed that surface roughness seems to be independent of the number of deposited layers. However, a higher removal degree is achieved as the thickness of the Fe_3_O_4_-TiO_2_ film increases in the studied range.

Finally, experiments reusing 1.5 µm:Fe_3_O_4_-TiO_2_ photocatalyst structures evidenced the stability of the fabricated thin films.

## Figures and Tables

**Table 1:** Average hydrodynamic size, polydispersity index and Z-Potential values for Fe_3_O_4_ NPs.

DLS Size (nm)	PDI	Z Potential (mV)
68.7	0.3	23.7

**Table 2:** Measurements of indirect and direct bandgap for different materials in this work.

Material	Indirect E_g_ (eV)	Direct E_g_ (eV)
TiO_2_	3.19 ± 0.05	3.61 ± 0.06
Fe_3_O_4_-TiO_2_	2.79 ± 0.05	3.31 ± 0.04
Fe_3_O_4_	0.93 ± 0.01	2.85 ± 0.06

**Table 3:** Root mean square, RMS (nm) of Fe_3_O_4_-TiO_2_ thin films.

Sample	Measurement	RMS (nm)
1 µm:Fe_3_O_4_-TiO_2_	1	25.6
	2	23.4
	3	21.5
	Mean ± s.d	23.5 ± 2.1
1.5 µm:Fe_3_O_4_-TiO_2_	1	20.2
	2	18.8
	3	15.9
	Mean ± s.d.	18.3 ± 2.2
2 µm:Fe_3_O_4_-TiO_2_	1	20.6
	2	24.8
	3	20.2
	Mean ± s.d.	21.9 ± 2.5

**Table 4:** Values of the apparent pseudo-first order rate constant using thin films with different thicknesses in the oxidation of SFX in water.

Sample	Thickness (µm)	k (10^−2^ min^−1^)
1 µm:Fe_3_O_4_-TiO_2_	0.97 ± 0.03	0.62 ± 0.004
1.5 µm:Fe_3_O_4_-TiO_2_	1.51 ± 0.04	1.03 ± 0.004
2 µm:Fe_3_O_4_-TiO_2_	1.99 ± 0.02	1.35 ± 0.003

## Data Availability

The datasets used and/or analysed during the current study are available from the corresponding author on reasonable request.
